# Evolution of prokaryotic SPFH proteins

**DOI:** 10.1186/1471-2148-9-10

**Published:** 2009-01-12

**Authors:** Markus Hinderhofer, Christina A Walker, Anke Friemel, Claudia AO Stuermer, Heiko M Möller, Alexander Reuter

**Affiliations:** 1 Department of Biology, Microbiology, University of Konstanz, Konstanz, Germany; 2 Department of Chemistry, NMR Spectroscopy, University of Konstanz, Konstanz, Germany; 3 Department of Biology, Neurobiology, University of Konstanz, Konstanz, Germany

## Abstract

**Background:**

The SPFH protein superfamily is a diverse family of proteins whose eukaryotic members are involved in the scaffolding of detergent-resistant microdomains. Recently the origin of the SPFH proteins has been questioned. Instead, convergent evolution has been proposed. However, an independent, convergent evolution of three large prokaryotic and three eukaryotic families is highly unlikely, especially when other mechanisms such as lateral gene transfer which could also explain their distribution pattern have not yet been considered.

To gain better insight into this very diverse protein family, we have analyzed the genomes of 497 microorganisms and investigated the pattern of occurrence as well as the genomic vicinity of the prokaryotic SPFH members.

**Results:**

According to sequence and operon structure, a clear division into 12 subfamilies was evident. Three subfamilies (SPFH1, SPFH2 and SPFH5) show a conserved operon structure and two additional subfamilies are linked to those three through functional aspects (SPFH1, SPFH3, SPFH4: interaction with FtsH protease). Therefore these subgroups most likely share common ancestry. The complex pattern of occurrence among the different phyla is indicative of lateral gene transfer. Organisms that do not possess a single SPFH protein are almost exclusively endosymbionts or endoparasites.

**Conclusion:**

The conserved operon structure and functional similarities suggest that at least 5 subfamilies that encompass almost 75% of all prokaryotic SPFH members share a common origin. Their similarity to the different eukaryotic SPFH families, as well as functional similarities, suggests that the eukaryotic SPFH families originated from different prokaryotic SPFH families rather than one. This explains the difficulties in obtaining a consistent phylogenetic tree of the eukaryotic SPFH members. Phylogenetic evidence points towards lateral gene transfer as one source of the very diverse patterns of occurrence in bacterial species.

## Background

The SPFH superfamily of proteins is a very diverse family of prokaryotic and eukaryotic membrane proteins that carry an evolutionarily conserved domain called the SPFH domain (named after the proteins **S**tomatin, **P**rohibitin, **F**lotillin and **H**flK/C) [1,2].

Members of this superfamily can be found in all domains including bacteria, archaea and eukaryotes. Although the conservation of the SPFH motif throughout all domains suggests that this motif is a primordial one with an important function [1,3], phylogenetic analysis has failed to provide additional support for the common ancestry of the SPFH superfamily [4], however this possibility is not ruled out. In addition, it has been suggested that the incongruency between species and protein trees of the SPFH2 members point towards sequence convergence [4]. There are however many reasons why trees produced from different family members can be inconsistent including base composition bias, mixture of orthologs and paralogs, the long-branch attraction artefact or lateral gene transfer [5-8].

Indeed, most known eukaryotic SPFH family members that have been investigated in-depth are involved in the scaffolding of specific detergent-resistant microdomains [2,9], suggesting that the SPFH domain may constitute a lipid recognition motif [3] regardless of whether or not all or every family member shares a common ancestor. However, the emphasis in those earlier studies was clearly put on eukaryotic SPFH proteins and only a limited number of prokaryotic SPFH family members were considered.

Only a few bacterial SPFH family members have been analyzed so far. The first was PH1510 in the Archaeum *Pyrococcus horikoshii *which has been shown to interact with the NfeD protein homolog PH1511, a serine protease [10]. The second was the Ybbk protein (Qmca) from *E. coli *[11]. Interestingly, a multicopy-Ybbk mutant conferred viability to the otherwise lethal deletion of *ftsH *protease and *htpX *protease, suggesting that YbbK might replace the function of those proteases by complementing their chaperone function. In addition, an interaction of YbbK with the FtsH protease has been shown [11].

The third, YuaG protein from *B. subtilis*, has been analyzed to some extent. The *yuaFGI *operon is dependent on the sigmaW factor which can be induced by different kinds of cellular stress, such as osmotic or alkaline shock or presence of toxic peptides [11]. Various sigmaW-controlled genes exist which provide their hosts with advantages in a competitive soil environment, e.g. by rendering them resistant to membrane-compromising antibiotics of other bacteria [12,13]. However, YuaG deletion mutants showed no growth defects under high salt or alkaline conditions [M. Hinderhofer, unpublished data] suggesting that YuaG is a factor for a specific but unknown stress resistance or that this resistance mechanism is of significance in a competitive environment only.

Studies using fluorescently-(GFP)-labeled YuaG, which localizes as a discrete and highly mobile foci within the plasma membrane, suggest that it might represent membrane microdomains in analogy to the situation in vertebrates [Peter Graumann and Felix Dempwolff, University of Freiburg; personal communication].

The fourth studied SPFH member SO1377 from *S. oneidensis *shows a pleiotropic phenotype with growth defects [14]. This deletion also has a strong effect on the iron metabolism in *S. oneidensis*.

Our own studies have shown that the SPFH member YqiK from *E. coli *is not induced by general cellular stress (e.g. osmotic or alkaline shock) and deletion mutants do not show growth defects in neither the standard or stress conditions mentioned above [M. Hinderhofer, unpublished data], suggesting again that YqiK might function under very specific yet unknown stress conditions. Overexpression of YqiK shows a marked effect on cell morphology. Bacteria that overexpress YqiK are larger than wild-type cells and contain opaque cellular inclusions suggesting that they might contain overproduced lipids [Helmut Plattner, University of Konstanz, unpublished data].

Despite the relative conservation of SPFH family member sequences and occurrence in higher eukaryotes, this consistency does not prevail in prokaryotes: while species with several SPFH family members exist, close relatives without family members exist as well. We performed a detailed search for SPFH family members in databases accessible to the public of completely sequenced prokaryotic genomes to address 3 issues: (1) what is the incidence and pattern of SPFH family members in prokaryotes; (2) is there an explanation of the very diverse occurrence of SPFH members, and (3) is there support for a common origin of SPFH proteins in bacteria and eukaryotes?

Our analysis confirmed that most, if not all, bacterial SPFH family members do indeed share a common ancestor, which is concluded from the strikingly conserved operon structures. The suggestion that the eukaryotic SPFH family members evolved from multiple – instead of one-bacterial SPFH family members would also account for the large sequence differences observed between the different eukaryotic SPFH families and the lack of a consistent phylogenetic tree for them.

The diverse pattern of occurrence together with conserved insertions in rather unrelated species strongly suggests lateral gene transfer.

## Methods

### Identification of SPFH proteins

To identify possible SPFH proteins, hypothetical sequences were scanned using CDART , smart  or Pfam . Following this initial scan, homologs of the identified proteins were searched for by multiple blast searches [15-17]. To ensure homologies, the cut-off value of 1E-25 was used for initial blast searches.

### Phylogenetic analysis

For the generation of phylogenetic trees, the most diverse sequences of each subgroup were aligned with ClustalW [18]. As some of the sequences showed a high sequence similarity, a criterion of at least 15% sequence divergence, as well as the origin from different genera was used for inclusion of sequences in this analysis. The complete list of all sequences included in the tree can be found in Additional file 1.

The alignment was used as input for the generation of phylogenetic trees with MEGA4 [19] (BLOSUM matrix and Neighbour-Joining algorithm). The statistical strength was assessed using bootstrap resampling.

Obtained trees were validated by repeating the analysis with other sequences of the subgroups. For the final grouping of all SPFH proteins into 12 subfamilies additional criteria like operon structures or domain architecture were also taken into account. In detail, SPFH1a and b were grouped together because they exhibit exactly the same domain structure, have a close similarity to the eukaryotic Stomatins and show the same operon structure. SPFH2a, b and c were grouped together because of the same reasons and their similarity to eukaryotic Reggies/Flotillins. SPFH3a and b were grouped together because they also share the same domain structure and because they are placed together in one operon. Finally, although SPFH11b lacks the predicted coiled-coil domain when compared to SPFH11a and both groups are separated in most trees they were grouped together because they are located in the same operon and both subgroups show a high similarity to the obtained consensus.

To obtain consensus sequences of each subgroup, all subgroup members were aligned using ClustalW 1.83 [18] and consensi were obtained with ESPript 2.2 [20].

In detail, for the generation of the consensus sequence of SPFH1 404 sequences were used, for SPFH2 127, for SPFH3a 247, for SPFH3b 248, for SPFH4 116, for SPFH5 36, for SPFH6 55, for SPFH7 26, for SPFH8 75, for SPFH8 32, for SPFH10 13, for SPFH11 14 and for SPFH12 11. The similarities to the obtained consensi were 43–80% for the SPFH1 family, 40–57% for the SPFH2 family, 59–82% for the SPFH3 family, 37–65% for the SPFH4 family, 79–95% for the SPFH5 family, 68–90% for the SPFH6 family, 79–81% for the SPFH7 family, 39–78% for the SPFH8 family, 53–76% for the SPFH9 family, 47–77% for SPFH10, 78–90% for SPFH11 and 55–74% for SPFH12.

To additionally verify that the separation of the subfamilies was correct, we used the HMMer program version 2.3.2  to create subgroup-specific HMMs and tested each of them against members of the other subgroups (see Additional file 1 and Additional file 2b). In detail, for the creation of the SPFH1a HMM, 257 sequences were use, for the SPFH1b HMM 95 sequences, for SPFH2a 63, for SPFH2b 36, for SPFH2c 18, for SPFH3a 247, for SPFH3b 248, for SPFH4 124, for SPFH5 36, for SPFH6 55, for SPFH7 24, for SPFH8 25, for SPFH9 30, for SPFH10 11, for SPFH11a 7, for SPFH11b 7 and for SPFH12 11.

The largest and most widespread group, the p-Stomatins, were denominated by SPFH1, followed by the p-Flotillins (SPFH2), which also showed a wide distribution among most prokaryotic families. The HflC/K proteins were denominated SPFH3 and the p-Prohibitins SPFH4. Additionally, the other small groups were numbered consecutively.

The highest and lowest E values of each subfamily were summarized in Additional file 2a.

### Primary sequence analysis

Hydropathy plots were created with WinPep 3.01 [21] and coiled-coil structures were predicted with algorithm described by Lupas et al. [22].

Analysis of GC and GC3 content as well as codon usage was performed using the DAMBE (Data Analysis in Molecular Biology and Evolution) program [23]. Codon usage of the respective organisms was retrieved from CMR (Comprehensive Microbial Resource, ).

For the calculation of Pearson's correlation coefficients, sequences were aligned and distance matrices were created with MEGA4 [19]. The distance matrices were then transformed into a vector and the Pearson's correlation coefficient was calculated with Excel.

## Results

### Most bacterial genomes contain at least one SPFH family member

Using known SPFH sequences as templates, we searched public databases for novel homologs. After scanning 980 bacterial genomes and filtering out redundancies (e.g. genomes from different strains of the same bacterial species), we constrained our analysis to the fully sequenced genomes of 497 species encompassing all bacterial phyla as well as archaea (see table 1, for a complete list see Additional file 1). Only 31 genomes that do not possess a single SPFH family member, mostly from the mollicutes and chlamydiae, were found (see Additional file 1).

**Table 1:** Distribution of the SPFH subfamily members within different bacterial phyla.

Bacteria (*)\SPFH subgroup	1a	1b	2a	2b	2c	3a/b	4	5	6	7	8	9	10	11a/b	12
Actinobacteria (42)	32	-	14	-	2	-	-	-	11	5	4	2	5	-	2
Aquificae (1)	-	1	-	-	-	-	-	-	-	-	-	-	-	-	-
Bacteroidetes/Chlorobi (27)	2	6	4	1	5	1	14	5	5	10	6	1	1	-	-
Chlamydiae (8)	-	-	-	-	-	-	-	1	-	-	-	-	-	-	-
Chloroflexi (8)	-	8	-	-	-	-	5	-	-	-	2	-	-	4	-
Cyanobacteria (31)	19	-	8	-	-	-	30	-	-	-	1	5	-	-	4
Deinococcus-Thermus (2)	1	-	1	-	-	-	1	-	1	-	-	-	-	-	-
Acidobacteria (2)	-	2	2	-	-	-	-	-	-	-	1	1	-	1	-
Firmicutes (73)	14	2	24	-	-	7	4	23	19	11	14	1	-	-	2
Fusobacteria (3)	3	-	1	-	-	-	1	-	-	-	-	-	-	-	-
Planctomycetes (3)	-	-	1	-	2	-	-	3	1	-	3	2	-	1	-
Alphaproteobacteria (87)	54	10	-	15	-	81	3	-	15	-	22	3	4	-	2
Betaproteobacteria (40)	33	25	-	3	-	38	6		-	-	12	7	1	-	2
Deltaproteobacteria (21)	12	12	2	-	3	10	4	-	-	-	15	2	3	1	1
Epsilonproteobacteria (15)	3	-	-	-	-	-	15	-	-	-	-	-	-	-	-
Gammaproteobacteria (113)	75	31	-	16	8	104	15	-	7	2	3	8	1	-	-
Spirochaetales (7)	3	-	-	-	-	5	2	-	-	-	3	1	-	-	-
Thermotogae (6)	5	-	-	-	-	5	-	1	-	-	-	-	-	-	-
**absolute numbers**	**256**	**97**	**57**	**35**	**20**	**251**	**100**	**33**	**59**	**28**	**86**	**33**	**15**	**7**	**13**

As the vast majority of all sequenced bacterial genomes belong to the phylum proteobacteria, we have subdivided this phylum into its respective classes.

### The bacterial SPFH family members can be divided in twelve subfamilies

Using the programs CDART , smart  and Pfam  we identified 1486 SPFH family members.

**Figure 1:**
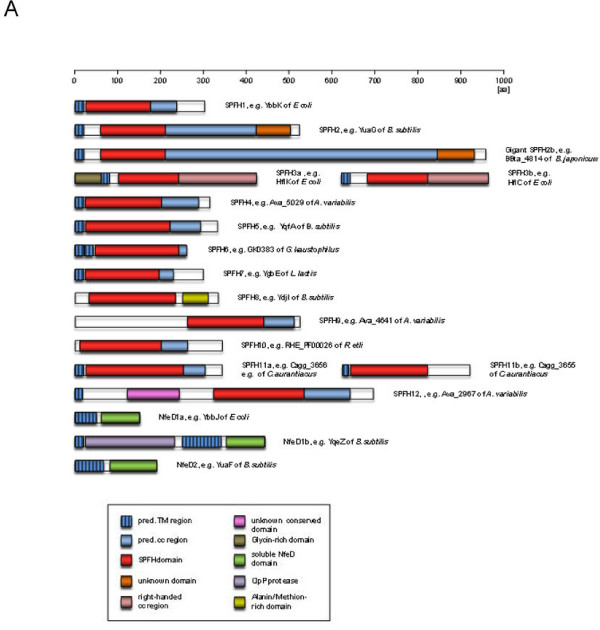
**Size, domain structure and phylogenetic tree of SPFH subfamilies**. This figure shows the upper half, for the full image please see Additional file 3. (a) Schematic representation of sizes and domain structure of all identified SPFH subfamilies, as well as the accompanying NfeD proteins. (b) Phylogenetic tree of prokaryotic SPFH proteins. The tree was created using the most diverse members of each subfamily, after alignment with ClustalW. Tree creation was performed with MEGA 4 (BLOSUM matrix and Neighbor joining algorithm, 1000 bootstrap replications).

Length and sequence conservation outside of the SPFH domain varied considerably (see Fig. 1a and Additional file 3) between the different family members and is consistent with the difficulties encountered when trying to compile a phylogenetic tree as mentioned by Rivera-Milla et al. [4]. However, a clear division into 17 subgroups was evident when we created our phylogenetic tree (Fig. 1b and Additional file 3, a rectangular tree has been included in Additional file 4). When incorporating other data such as operon structures (see methods), these subgroups could be grouped into 11 subfamilies (SPFH1-4 and 6–12). 38 sequences, less than 3% of all sequences considered, could not be included due to insufficient statistical support. We created additional HMMs for each subgroup and tested each of them against members of the other subgroups to ensure that the subfamilies are indeed separated from each other. The cut-off values which ensure membership in a given family have been summarized in Additional file 2b and can be seen in detail in Additional file 1, sheet 5–21.

In addition, a new subgroup (SPFH5) was described and added as a SPFH family, thus a total of twelve SPFH subfamilies were identified in the end.

The difficulty in identifying the new SPFH5 family as members of the SPFH superfamily was largely due to the relatively low sequence identity of their SPFH domain to the canonical SPFH motif. It was formerly denoted as a separate HMM called COG4864. This SPFH5 subgroup (see Additional file 2a) holds one of the highest E values to the generic SPFH hidden Markov Model defined by Pfam entry PF01145 (HMM).

**Figure 2:**
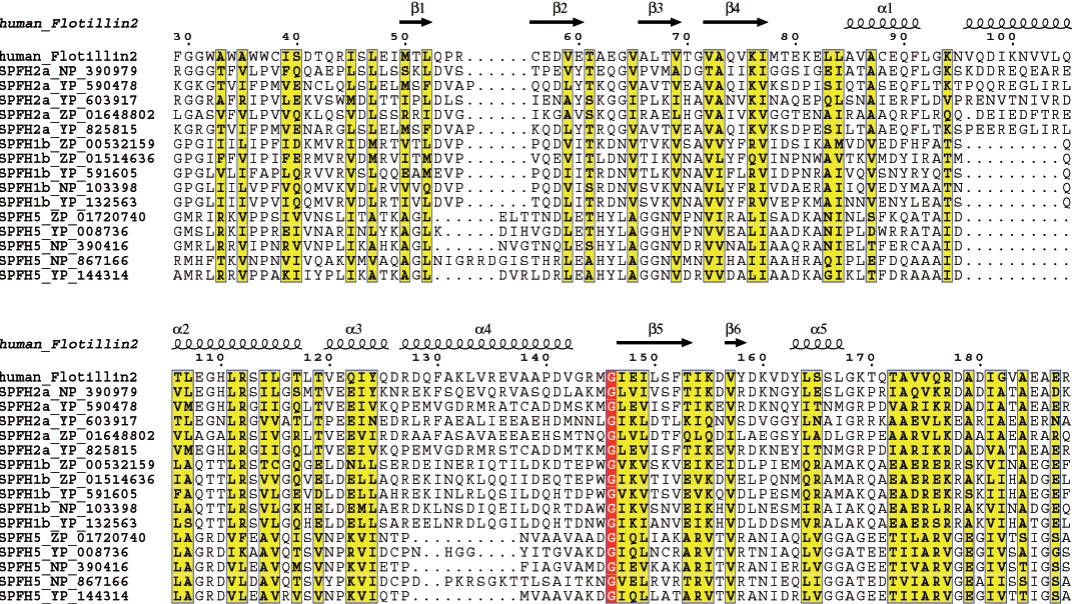
**Comparison of SPFH1,2 and 5 subgroup members**. Partial alignment and secondary structure annotation between SPFH1,2 and 5, using the already known 3D structure of Flotillin2 (PDB ID: 1WIN). The alignment of the SPFH domains of different SPFH1,2 and SPFH5 members reveals a continuum of similarity between both groups, suggesting that SPFH5 members belong to the SPFH superfamily.

**Figure 3:**
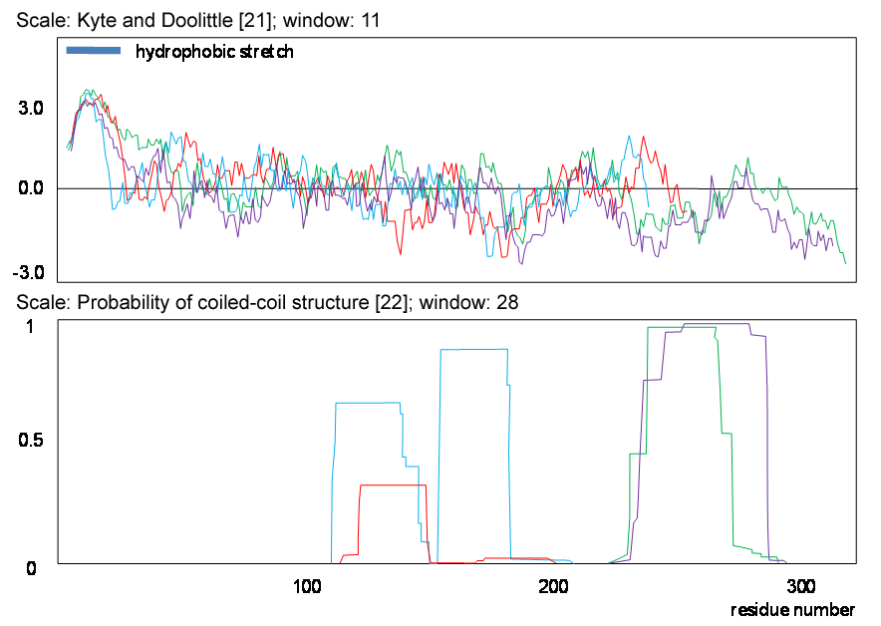
**Hydropathy plots and coiled-coil prediction of SPFH1b and SPFH 5 member proteins showing similar general structure**. (A) Superimposed hydropathy plots and (B) coiled-coil predictions; purple: SPFH5 of *Bacillus. subtilis *(NP_390416); green: SPFH5 of *Thermus thermophilus *(YP_144314); blue: SPFH1b of *Pseudomonas entomophila *(YP_605957); red: SPFH1b of *Chloroflexus aggregans *(ZP_01514636).

When analyzing all available data, especially the sequences from rather exotic species (e.g. *Algoriphagus sp*. PR1), a continuum of similarity to the SPFH1 and 2 subgroups could be seen that strongly suggests their relation to the SPFH superfamily (Fig. 2). In addition, structural predictions (Fig. 3), as well as a conserved operon structure (see section on **Conserved operon structures of most SPFH family members**) compared with those of well-known SPFH family members further emphasizes that these SPFH5 proteins indeed belong to this family. In detail, the classic SPFH members contain at least 1 hydrophobic stretch that most likely represent transmembrane domains at the N terminal of the protein and a predicted coiled-coil domain necessary for oligomerization located in the C terminal section. The SPFH5 subgroup shares this predicted structure (Fig. 3). The hydropathy plots also share their basic structure (Fig. 3). To comply with the nomenclature introduced by Tavernarakis et al. [1], we denoted the different subgroups SPFH1 to SPFH 12 (Fig. 1a,b and Additional file 3).

Only 2 of the 12 subfamilies seem to be soluble (SPFH8 and 9), all other groups contain predicted transmembrane regions at their N terminus. The distribution of the SPFH family members among the bacterial phyla is shown in table 1. Because the vast majority of all sequenced bacterial genomes belong to the phylum proteobacteria, we have subdivided this phylum into its respective classes [24].

### SPFH subfamily description

The p-Stomatins [1] represent the largest and most widespread subgroup and are denoted as SPFH1 proteins. On the amino acid level, representatives show a high similarity to the eukaryotic Stomatins (e.g. 29% identity and 67% similarity between human Stomatin2 and YbbK in *E. coli*). In general, they are membrane proteins between 28 to 47 KDa and their hydrophilic part is oriented towards the cytoplasm [11].

The eukaryotic Stomatins and the bacterial SPFH1 proteins form oligomeric complexes [11,25]. According to primary structure, the SPFH1 subfamily can be further divided into 2 subgroups, SPFH1a and 1b.

The SPFH2 subfamily (the p-Flotillins) shows a high degree of similarity with the eukaryotic Flotillins/Reggies (e.g. the identity between human Reggie1/Flotillin2 and YuaG of *B. subtilis *is 37% and the similarity 74%).

Although their relatives can be found in every higher eukaryote, they are restricted to about 20% of all bacterial genomes investigated. This SPFH2 subfamily can also be further divided into 3 subgroups according to their primary sequence.

Remarkable members of SPFH2b subgroup are the giant SPFH2 proteins that are characterized by a strongly expanded coiled-coiled region. These giant SPFH2 proteins are about 800–1000 aa in length.

The SPFH3a/b subfamily contains the HflK/C proteins. This subfamily is the second largest SPFH subfamily in bacteria, however, it is absent from archaeal genomes (and from eukaryotes). The HflK/C oligomers have been shown to interact with the FtsH protease [26] much like the YbbK (SPFH1a). However, the SPFH3 proteins are oriented towards the periplasm [26] in contrast to YbbK which is oriented towards the cytoplasm.

The prokaryotic Prohibitins have been denoted as the SPFH4 subfamily. It is the most diverse subfamily with regard to its primary structure of member proteins. No bacterial members of this group have been investigated so far, however, the closely related eukaryotic Prohibitins have been studied intensely. They are located in the inner membrane of mitochondria and have been shown to interact with the FtsH protease [27] there. Their function has been described as chaperoning of mitochondrial membrane proteins [28,29]. This suggests that prokaryotic Prohibitins might also function as membrane protein quality control, a function that is required especially during osmotic changes and/or other rapid milieu changes.

The SPFH5 subfamily is mainly found in chlorobi, firmicutes and planctomycetes. As mentioned above, their SPFH domain is poorly conserved; therefore most programs do not recognize them as members of the SPFH family. However, compared to SPFH1b, their general primary structure and conserved operon structure strongly suggest that these proteins belong to the SPFH superfamily. As of yet, no genome has been found that contains both a SPFH1b and SPFH5 together and their operon structure is similar, hence it seems likely that SPFH5 replaces SPFH1b in the relevant bacterial species. The SPFH5 member YqfA from *B. subtilis *has been reported to confer resistance against toxic peptides [13].

The other subfamilies (6–12) are rather small and contain no documented member proteins. The SPFH8 and SPFH9 families show no predicted transmembrane domains and are therefore most likely soluble.

### Evidence for lateral gene transfer

A striking observation concerning the distribution of SPFH family members among prokaryotes is the fact that some organisms have several SPFH family members, while other even closely related species do not even possess one. Even organisms belonging to the same genus differ remarkably in their content of SPFH family members. *B. halodurans*, for instance, harbours 4 SPFH family members (namely, the BH3500, BH3154, BH3155 and BH1357 proteins), whereas the closely related *B. licheniformis *contains only 1 SPFH family member (the BLi02729 protein). A distribution like this can hardly be explained by selective gene duplication and loss events, rather suggests lateral gene transfer as major reason for these distribution patterns.

In addition, some SPFH families are rather equally distributed among the bacterial phyla (e.g. SPFH 1,2,4,8 and 9), while other subgroups seem to be restricted almost exclusively to certain phyla (e.g. SPFH 3a/b is restricted to the proteobacteria with only a few exceptions) and only a very few other unrelated bacterial species contain a member of these subgroups. To explain this pattern of occurrence we either have to propose that the SPFH3 subgroup members where originally included in the other genomes and were eventually lost over time, or we have to consider other mechanisms leading to this distribution, such as lateral gene transfer. However, quite small subfamilies exist containing only a very few members (e.g. SPFH9-12) which are distributed throughout a wide variety of unrelated species in different phyla that also cannot easily be explained by gene duplication and/or gene loss.

**Figure 4:**
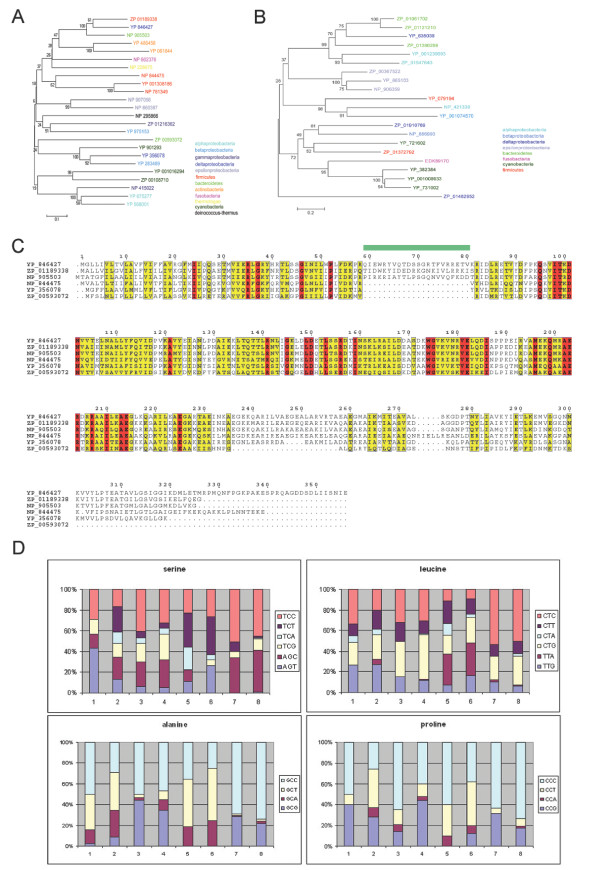
**Evidence for lateral gene transfer**. (a) Phylogenetic tree of SPFH1 member proteins (BLOSUM matrix and neighbor joining algorithm, 1000 bootstrap replications). The ZP_01189338, YP_846427 and NP_905503 proteins, originating from bacterial species belonging to different phyla, are more closely related to each other than to SPFH1 proteins from bacterial species originating from the same phylum. (b) Phylogenetic tree of SPFH4 member proteins (BLOSUM matrix and neighbor joining algorithm, 1000 bootstrap replications). The ZP_01910769 gene from the deltaproteobacterium *P. pacifica *shows a closer evolutionary relationship to proteins from other phyla than to SPFH4 proteins from other deltaproteobacteria. (c) A common insertion in SPFH1 members in only distantly related species suggests lateral gene transfer. A 22 aa Insertion (aa 236–257) can be found in a few distantly related species, but in more closely related species it is missing, thereby suggesting lateral gene transfer. Compare YP_846427 of *Syntrophobacter fumaroxidans *(with the insertion) and YP_356078 of *Pelobacter carbinolicus*, both belonging to proteobacteria; compare ZP_01189338 (with insertion) of *Halothermothrix orenii *and NP_844475 of *Bacillus anthracis*, which belong to firmicutes; compare NP_905503 (with insertion) of *Porphyromonas gingivalis *and ZP_00593072 from *Prosthecochloris aestuarii *which belong to bacteroidetes. (d) Codon usage of the NP_905503 gene (1) compared with codon usage of total coding sequences in *P. gingivalis *(2), for the YP_846427 gene (3) compared with total coding sequences of *P. fumaroxidans *(4), of the ZP_01189338 gene (5) compared with total coding sequences of *H. orenii *(6) and of the YP_144313-15 operon (7) compared with *T. thermophilus *total coding sequences (8). Synonymous codons are shown to the right of each amino acid and color-coded to match the percent usage indicated by the bars.

Several approaches exist for the detection of lateral gene transfer [5,6]. Among them are phylogenetic methods like the unexpected ranking of sequence similarity among homologs where genes from an organism show the strongest similarity to a homolog in a distant taxon. Indeed, the SPFH1 proteins of some species have a closer evolutionary relationship with sequences in other genera than with their closer relatives. For example, the SPFH1 gene YP_846427 in *Syntrophobacter fumaroxidans *(deltaproteobacteria) shows 58% identity to the SPFH1 protein ZP_01189338 in the distantly related *Halothermothrix orenii *(firmicutes), but only 34% identity to the YP_356078 protein in the closely related *Pelobacter carbinolicus *(also deltaproteobacteria, Fig. 4a).

A similar observation can be made regarding most other SPFH subgroups, e.g. the SPFH4 subgroup, where the ZP_01910769 protein from *Plesiocystis pacifica *(deltaproteobacteria) shows a much closer relation (identity 30%) to the EKD89170 (= ZP_02273092) protein from *Fusobacterium nucleatum *(fusobaceria) than to the ZP_01462952 protein from its close relative *Stigmatella aurantiaca *(deltaproteobacteria, 20% identity, Fig. 4b).

Moreover, we identified several SPFH family members belonging to the SPFH1a subgroup that contain a 22 aa insertion in the same position just prior to the SPFH domain. Interestingly, these three proteins belong to organisms that are only very distantly related, e.g. *H. orenii *(phylum firmicutes), *P. gingivalis *(phylum bacteroidetes) and S. fumaroxidans (phylum proteobacteria). Closer relatives to each of them have homologs without this insertion (Fig. 4c). For example, *H. orenii *contains the YbbK protein with the above mentioned insertion, whereas its close relative *B. anthracis *does not. Likewise, the closely related *B. subtilis *lacks the *ybbK *completely. Although the overall protein sequence is remarkably similar among these three genes, the insertion shows almost no conservation at all, indicating that the inserted sequence may not be essential for the protein function suggesting that no evolutionary pressure acts on sequence conservation.

Other approaches used to detect lateral gene transfer include the analysis of nucleotide composition or codon usage biases [5]. Laterally transferred genes often show a significantly higher or lower GC content, in total as well as at the third codon position (GC3) due to the fact that they originated in an organism with a different GC content [5]. However, when analysing the GC content of the above mentioned proteins, only the NP_905503 protein of *P. gingivalis *showed a significantly higher GC3 content relative to the GC3 content of its whole genome (YP_846427: GC 57.4%, GC3 76.4% compared with coding GC 60.2% and GC3 75.5% generally found in *S. fumaroxidans*; ZP_01189338: GC 40.7%, GC3 38.8 compared with GC 40.8% and GC3 32.9% generally found in *H. orenii*; NP_905503: GC 52.5%, GC3 64.5% compared with GC 47.6% and GC3 50.0% generally found in *P. gingivalis*).

Another possible candidate gene for lateral transfer is the SPFH5 member YP_144314 from *Thermus thermophilus*. Although *Thermus termophilus *has an extremely high GC content, the whole operon (the YP_144313 gene of unknown function, the YP_144314 SPFH5 gene and the YP_144315 NfeD gene) closely resembles relatives in firmicutes which display a very low GC content (most SPFH5 members are found in firmicutes and planctomycetes). The whole operon has a GC (69.60%) and GC3 (92%) content that closely resembles the one found in all *Thermus *coding sequences (GC: 69.35%).

Codon usage can also hint towards whether a gene has become laterally transferred or not. Organisms do not use all synonymous codons with the same frequency and often show particular preferences for one of the several codons that encode the same amino acid. Laterally transferred genes often still show the codon preference of the organism they originated from [5]. Codon usage was therefore analyzed for the above mentioned genes (NP_505503, NP_846427, ZP_01189338 and the YP_144313-15 operon). Fig. 4d illustrates that all except the NP_905503 gene showed only slight deviations from the overall codon usage of their respective species, which fails to provide additional support for lateral gene transfer in these cases. However, the NP_905503 gene revealed modest differences, especially in the codon usage for proline, lysine, threonine, glutamine, serine or valine (Fig. 4d).

### Conserved operon structures of most SPFH family members

Recently the SPFH1 subgroup member PH1510 of the archaeon *Pyrococcus horikoshii *was reported to interact with the NfeD protein homolog PH1511, a serine protease [10]. Moreover, PH1510 forms an operon with PH1511 thereby sharing the regulation and expression with this NfeD homolog. The SPFH subgroups sequences are very diverse therefore common ancestry is difficult to assess. A conserved operon structure would clearly hint towards common ancestry. We therefore examined the genetic vicinity of the known SPFH proteins for conservation of operon structures and for the presence of possible other NfeD homologs.

As expected, most (229 from 353) of the other members with close similarities in the SPFH1 subgroup such as the YbbK and the SO4129/SO4128 proteins share an operon with a NfeD homolog (in this case, YbbJ and SO4130, respectively), thereby indicating common origin for these subgroup members.

These NfeD homologs form a protein family that we denoted NfeD1, with SPFH1a members paired to NfeD1a and SPFH1b members paired to NfeD1b subfamily members. In some species, a gene duplication event has obviously occurred replicating the SPFH1 member which results in operons of the NfeD1a-SPFH1a.1-SPFH1a.2 type.

**Figure 5:**
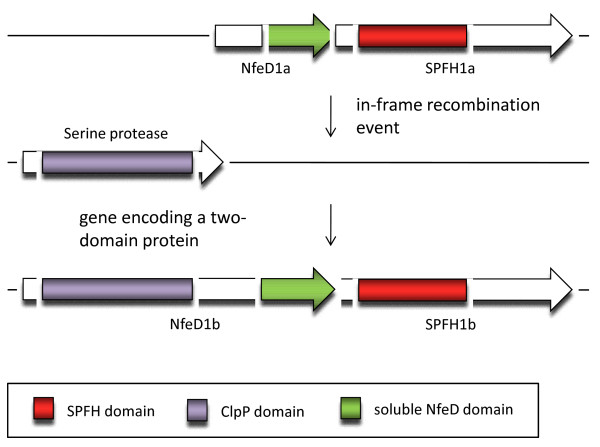
**Hypothetical scenario of a recombination event that created the NfeD1b protein**.

Interestingly, the NfeD1b proteins differ from NfeD1a insofar as that they contain a ClpP protease domain at their N terminus, thereby indicating that the NfeD1b-SPFH1b operon may have arisen from a recombination event that translocated the NfeD1a-SPFH1a operon directly adjacent to a ClpP protease (Fig. 5). In an intergenomic comparison, the distribution of both operons seems random; sometimes, both can be found in one genome whereas in other genomes, only one can be identified. In some genomes, no NfeD could be found in the vicinity of the SPFH1 members. It is difficult to assess whether there are no partner NfeD proteins present or whether they have escaped detection, probably because they are not located in the same operon as the SPFH1 genes and/or to a larger degree they have diverged from the consensus sequence.

As mentioned above, the search for new SPFH family members revealed a completely new subgroup. The SPFH5 group shows a relatively low sequence similarity to the other family members. However, these proteins (e.g. YqfA in *B. subtilis*) clearly form an operon with another protein (e.g. YqeZ in *B. subtilis*) which is a PH1511 homolog (including the ClpP protease motif) thereby not only strengthening the membership of the YqfA (SPFH5) family in the SPFH protein superfamily, but also strongly suggesting that the whole SPFH5 subgroup evolved from the SPFH1b subgroup.

We extended our search of NfeD homologs to the SPFH2 subgroup, the prokaryotic Flotillin/reggie homologs. The SPFH2 group members (e.g. *yuaG *from *B. subtilis*) form an operon with another gene, namely *yuaF *(and, only in *B. subtilis*, also with *yuaI*). Whereas no significant sequence similarity between NfeD and YuaI was found, low similarity could be detected between NfeD and some YuaF proteins. Indeed, YuaI belongs to the family of GCN5-related acetyl transferases.

Despite low similarity at the amino acid level, structural predictions and hydrophobicity plots resulted in remarkably similar characteristics for known NfeD members and YuaF, thereby suggesting that YuaF is a lowly conserved NfeD homolog. In detail, most NfeD homologs have a length of 140–150 aa and consist of 3 hydrophobic stretches at their N terminal region followed by a short hydrophilic stretch and a soluble all-beta domain whose structure (PH0471 from *P. horikoshii*) has been recently solved [PDB ID: 2EXD]. This general structure can also be seen in the *yuaF *gene. However, structural predictions have a high degree of uncertainty and therefore conclusions drawn only on predictions have a weak basis.

**Figure 6:**
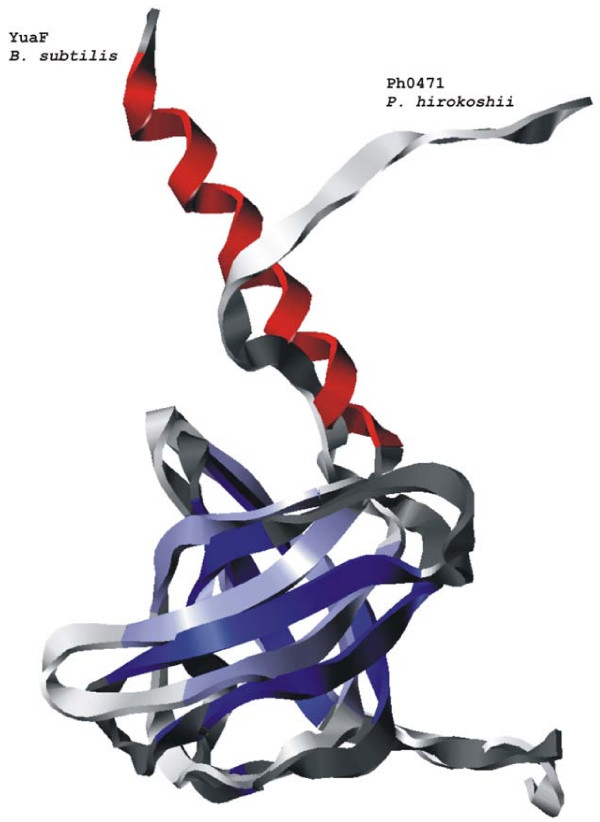
**Comparison of YuaF and PH0471 structures**. Superposition of the C-terminal domain of YuaF from *B. subtilis *as determined by NMR (PDB entry 2K12) [26] with the NfeD homolog PH0471 from *P. horikoshii *(PDB code: 2EXD). β-sheets are blue, the N-terminal α-helix of YuaF is red. Random coil regions are yellow. Both protein domains display a striking similarity in their β-barrel cores.

Recently, we have determined the 3D structure of YuaFs soluble C-terminus by NMR spectroscopy [30]. Indeed, the solution structure reveals a striking similarity to a known, SPFH-associated NfeD protein, PH0471, from the archaeon *P. horikoshii *[PDB ID: 2EXD] [31], despite very low sequence identity (21%) (Fig. 6).

In addition, we have determined NMR chemical shifts (BMRB entry 15664) in the soluble C-terminus of YqiJ of *E. coli*, the gene of which is found in one operon together with the reggie-like gene yqiK. Indeed, the deviations of H^α^, C^α^, C^β^, and CO chemical shifts from values found in random coil structures [32] strongly support the secondary structure content found by prediction algorithms. Both the structure of YuaF, as well as the chemical shift assignments of YqiJ further support our hypothesis that all SPFH-associated NfeD proteins share a common structure and thus have a common origin.

In the SPFH2 subgroup, the operon structure is therefore also conserved insofar as a putative NfeD homolog (YuaF/YqiJ/Syc1604_d) shares an operon with one (YuaG/YqiK) or two (Syc1607_d/Syc1608_d) SPFH2 family members. In cyanobacteria, a gene duplication of the SPFH family members similar to the one in the SO4130/SO4129/SO4128 operon obviously has occurred.

**Figure 7:**
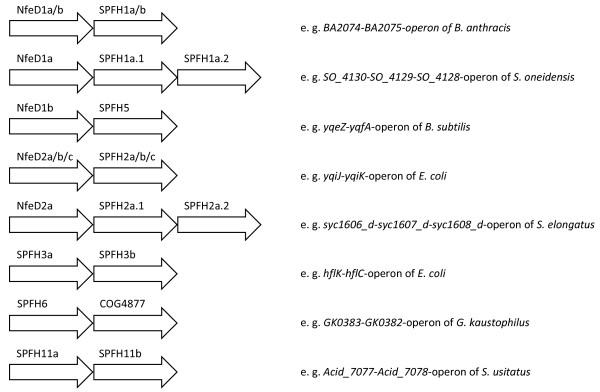
**Representative operon structures of SPFH and accompanying NfeD genes**. In some cases, there may be additional genes organized inside these operons that have been left out for clarity.

Figure 7 summarizes all operonic combinations of the different families. In correspondence with the SPFH1a and the SPFH2a-c subgroups, the corresponding NfeD proteins also form a strikingly similar phylogenetic tree with corresponding subgroups (NfeD1a and NfeD2a-c, see Additional file 5).

To quantify this, Pearson's correlation coefficients were calculated. Whereas the overall correlation between the SPFH1 and 2 trees and their respective NfeD partners was 0.495, the individual correlations between the different subgroups varied between 0.255 (SPFH1a and NfeD1a), 0.352 (SPFH2c and NfeD2c), 0.511 (SPFH2b and NfeD2b) and 0.911 (SPFH2a and NfeD2a), underlining the fact that they have co-evolved together with their SPFH partners. The NfeD1b subgroup that accompanies the SPFH1b subgroup has been omitted in this analysis because the ClP protease domain present in these NfeD proteins prevents proper sequence alignment.

The other SPFH subgroups have no neighbouring putative NfeD homologs and none of their accompanying genes show identifiable sequence or structure conservation to other genes that share operon structures with other known SPFH family members.

## Discussion

### Most bacterial genomes contain at least one SPFH family member

In this study, we have analyzed 497 fully sequenced bacterial genomes and identified 1486 SPFH superfamily members from 466 genomes. Phylogenetic analysis revealed 17 subgroups which could, when taking other criteria such as operon structures into account, be grouped into 12 subfamilies. The four largest subfamilies which correspond to the already mentioned p-Stomatins, p-Flotillins, HflC/Ks and p-Prohibitins account for roughly 75% of all sequences identified and were denoted SPFH1-4, the additional 8 subgroups were numbered sequentially. Of those smaller subgroups, only the SPFH11 subgroup revealed a split into 2 separate groups in the phylogenetic tree. However, as the SPFH11a and b members invariantly occur adjacent to each other and are organized into one operon, they most likely originated in a gene duplication and were grouped together to reflect this.

SPFH member proteins were not found in 31 genomes, although we cannot rule out the possibility that they only escaped our notice due to extremely low sequence similarity.

Interestingly, most of those bacterial species without any identifiable SPFH family member are endosymbiontic or endoparasitic bacteria (e.g. the mollicutes or the chlamydiae) whose surrounding milieu is mostly isoosmotic or constant.

One of the more intensely investigated members, the SPFH2a YuaG gene in *Bacillus subtilis*, is part of the sigmaW regulon, a regulon that is activated in conditions of cellular stress like high salt, alkaline shock and ethanol stress [33], conditions that arise, for example, during diurnal changes in a soil environment. Moreover, various sigmaW controlled genes render expressing bacteria resistant to membrane-compromising antibiotics in other bacteria [13]. Our investigations showed no growth defects in YuaG deletion mutants under conditions of cellular stress [M. Hinderhofer, unpublished data]. However, there may be no knock-out phenotype as laboratory strains tend to loose such genes and other parts of the protective machinery may have already been lost or conditions may not be competitive. It may therefore be perfectly possible that SPFH proteins confer a slight, but not vital advantage in the cellular reaction to rapidly changing milieus, which would make them good candidates for lateral gene transfer, much like antibiotic resistance genes.

### Evidence for lateral gene transfer

It has been stated earlier that the discrepancy between species and gene trees in bacterial SPFH2 proteins questions the relatedness of bacterial members to metazoan Flotillins [4]. However, there are many reasons that could produce such a result and mask phylogenetic relationships, e.g. mixture of orthologs and paralogs, unequal rates of evolution, the long branch attraction artefact or lateral gene transfer [5-8]. Lateral gene transfer has often been identified in bacterial genomes as being one of the major sources of genetic diversity in prokaryotes [34-36].

Indeed, the diverse pattern of SPFH protein occurrences even in closely related bacterial species suggests a remarkable amount of lateral gene transfer also in case of the SPFH proteins. Phylogenetic evidence such as a strong incongruency between the species and the SPFH protein trees among prokaryotes supports this view. Detection of three SPFH1 family members from different genera with a similarly sized insertion at the same position shows that the SPFH1 sequences of some species have a closer evolutionary relationship with sequences in other genera than with their closer relatives. This suggests a common ancient origin and that lateral gene transfer has occurred among unrelated genera. Interestingly, the overall protein sequence among these three genes is remarkably similar, indicating evolutionary pressure leading to sequence conservation. This is not true for the above mentioned insertion which seems to be quite irrelevant for protein function hence no evolutionary pressure for sequence conservation is evident.

Other approaches used to detect laterally transferred genes such as differences in GC content or codon usage only showed slight deviations from the respective species averages for most genes investigated, indicating that the transfer event happened a long time ago and characteristics have already started to conform to their new genomic surroundings. Only the NP_905503 gene from *P. gingivalis *showed modest deviations in GC3 content and in the coding preferences for some amino acids which supports the theory that this gene has been laterally transferred.

This suggests that at least part of the complex pattern of SPFH protein distribution probably originated from lateral gene transfer rather than classical gene duplication and/or loss events. The lack of observable differences in GC content and codon usage in most genes investigated indicates that the transfer event seems to have happened quite some time ago.

### Operon structure

We have shown that most of the SPFH1 and 5 members are located in an operon together with a NfeD protein homolog. The SPFH2 family members are also located in an operon together with another protein which shows a very weak similarity to known NfeD proteins (e.g. YuaF from *B. subtilis *exhibits only 21% sequence identity with PH0471 from the archaeon *P. horikoshii*). The structure of those SPFH2 accompanying genes however is remarkably similar to the structure of known NfeD genes, thereby suggesting that these genes (e.g. YuaF) are minimally conserved NfeD homologs. In addition, phylogenetic trees of SPFH1 and 2 genes are remarkably similar to trees of their NfeD partner genes (NfeD1 and 2, see Additional file 5), indicating that both proteins evolved together and also have been transferred laterally together.

Occasionally, in some genomes SPFH1 or SPFH2 family members were found that are closely related to those groups, but that do build single gene cistrons lacking an identifiable NfeD (in 50 genomes we found an SPFH1 without identifiable NfeD1 and in 17 genomes an SPFH2 without NfeD2). However, their close similarity to the ones in operons with an NfeD part suggests that the first were derived from the second by a gene duplication and subsequent loss of the NfeD part. Furthermore it is possible that they interact with a known NfeD partner from another operon or with one from a neighbouring operon that, due to low sequence conservation, escaped our notice.

No NfeD homolog could be identified for the other (SPFH3,4, 6–12) subfamilies.

However, the sequence similarity between SPFH4 and the eukaryotic Prohibitins suggests a similar topology (that is, they most likely are oriented towards the periplasm), much like the SPFH3 subfamily, although this has not yet been tested,. With some rare exceptions, SPFH4 family members do not occur together with SPFH3 family members in the same genome, which suggests that the SPFH4 proteins may replace the SPFH3 proteins. Therefore, taking into account the relatively large sequence similarity to the SPFH1 and SPFH3 subgroups, a scenario by which the SPFH4 operon developed from the SPFH1 operon through deletion of the NfeD protein homolog is possible. The SPFH3 operon would then originate from the SPFH4 operon by a simple duplication event. This is further supported by the fact that the SPFH1 family member YbbK in *E. coli *(together with its NfeD homolog YbbJ) has been shown to interact (as multimers) with the FtsH protease [11], a behaviour which has also been shown for the SPFH3 (HflK/C) multimers [26] and the eukaryotic Prohibitin multimers [37].

Although this interaction has not yet been shown for the SPFH4 proteins, the sequence and predicted structural similarity to the eukaryotic Prohibitins proteins and the SPFH3 (HflK/C) proteins suggests a similar behaviour for these genes and therefore would additionally point towards a common origin for SPFH4 and SPFH1,2,5 and 3 proteins.

Presently several interaction partners with eukaryotic SPFH family members have been identified [38,39], although no NfeD proteins have been found in eukaryotes yet.

As is reflected in their denomination as p-Prohibitins, the prokaryotic SPFH4 members show a very close similarity to eukaryotic Prohibitins. As mitochondria are thought to have developed from alphaproteobacteria, it is intriguing to speculate that the eukaryotic Prohibitins (that are located in the inner membrane of mitochondria) entered the eukaryotic genome via such endosymbiosis.

At this point we cannot find any additional data corroborating common origin for the other subgroups. We cannot however rule out the possibility that these SPFH subgroups also interact with one of the known or even unknown NfeD homologs although they do not share the same operon. It may also be possible that other, weakly conserved NfeD homologs, are contained in the regulon encompassing the other SPFH genes that escaped our search.

### Common ancestry of most SPFH family members

**Figure 8:**
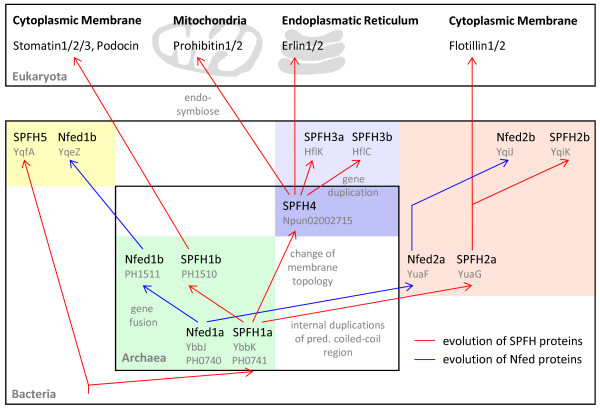
**Model of SPFH and NfeD coevolution**. Hypothetical model of prokaryotic SPFH and NfeD protein coevolution, based on the phylogenetic relationships and operon structures.

Weak sequence similarities alone have been questioned to be a good basis to infer common ancestry upon [4]. However, we propose an evolutionary relationship for the SPFH families and their putative NfeD partners as shown in Fig. 8 in conjunction with other data such as a common operon structure and functional aspects. According to this, a proto-SPFH together with a proto-NfeD gave rise to the SPFH1 (p-Stomatins) family (and, maybe directly or via SPFH1, to the SPFH5 subfamily). This SPFH1 family might then be the origin of the SPFH2 family (prokaryotic Flotillin/reggie homologs) and, by loss of the NfeD part and duplication of the SPFH gene, of the HflC/K (SPFH3) proteins. This step may have arisen via the SPFH4 (p-Prohibitins) proteins. Although the topology (orientation towards the cytoplasm or towards the periplasm) of the SPFH4 members is still unknown, their similarity to both the SPFH3 and the eukaryotic Prohibitins suggests that they may be oriented towards the periplasm, as well. The SPFH5 group develops either by duplication of the SPFH1 operon and fusion of the NfeD gene with a protease or directly from the proto-SPFH. The origin of the other groups still remains unclear. However, those groups are mostly rather small, and most of them show a close similarity to one of the main subgroups, therefore at least some of them might be considered as special subgroups of one of the larger groups.

### Origin of eukaryotic SPFH members

Earlier investigations mentioned the lack of regions of synteny between the eukaryotic SPFH family members, the fact that the sequence similarity between the different groups (Stomatins, Prohibitins and Flotillins/Reggies) is restricted to only a few amino acids, and the lack of a consistent phylogenetic tree obtained when comparing their sequences including prokaryotic ones, suggesting that those families might have evolved by convergent evolution [4]. In addition, it has been stated that the discrepancy between species and gene trees in bacterial SPFH2 proteins questions the relatedness of the bacterial members to the metazoan Flotillins [4]. This question is difficult to assess, as there is no common accepted phylogeny for bacterial groups. In addition, horizontal gene transfer plays a major role in bacteria which further masks phylogenetic relationships [34-36]. The evidence of a high rate of horizontal gene transfer in the case of the bacterial SPFH proteins and their accompanying NfeD partners as shown here would therefore explain this discrepancy even if an accepted model of the phylogenetic relationships between the prokaryotic phyla existed. In addition, sequence length, which differs between prokaryotic and eukaryotic family members, is not a good indicator for or against common origin, as in the case of the giant SPFH2s. Even within a group clearly related proteins can be found that differ greatly in length, mostly because the predicted coiled-coil region is elongated (see Fig. 1a and Additional file 3).

Although we have no direct evidence that the prokaryotic and the metazoan SPFH families are related, some facts are intriguing and speak against convergent evolution and for common origin.

First, we could show that at least 4 (or 5) of the major prokaryotic SPFH families (that encompass roughly 75% of all found bacterial SPFH proteins) share a common origin. Therefore, at the bacterial level, the p-Stomatins (SPFH1, and SPFH5), p-Flotillins/Reggies (SPFH2) and p-Prohibitins (SPFH4 and, most likely belonging to them, SPFH3) are clearly related not only due to their sequence identity, but also due to their common operon structure and functional aspects. In the case of the Prohibitins, a functional link between the prokaryotic SPFH3 and 4 families and the eukaryotic Prohibitins exists in form of their interaction with the FtsH protease and their topology hence common origin is rather likely. For the Flotillins and the Stomatins this functional link is still missing, although priliminary observations speak of a membrane distribution for prokaryotic Flotillins/Reggies that is strikingly similar to that in eukaryots [Graumann and Dempwolf, personal communication].

In light of these findings it becomes unrealistic to hypothesize that in eukaryotes, as with prokaryotes, three major families should also have developed purely by convergent evolution which share the same basic features and whose member proteins, when included in the phylogenetic analysis, sort into the correct subfamilies of their prokaryotic counterparts (e.g. eukaryotic Flotillins sort into the SPFH2 subgroup without any tweaking or manual adjustment).

Similarities in the p-Stomatins (SPFH1) to the eukaryotic Stomatins, the HflC/K (SPFH3) and SPFH4 proteins (including function and their membrane topology) to the eukaryotic Prohibitins, and the SPFH2 proteins to the eukaryotic Flotillins/Reggies would then suggest that the origin of the three eukaryotic SPFH families lies in the three major prokaryotic SPFH1/5, SPFH2 and SPFH3/4 groups. Another possible scenario includes the evolution of the three eukaryotic families from only one prokaryotic family.

However, such a scenario would either implicate that the prokaryotic counterparts of these eukaryotic SPFH families have evolved independently, or that at least the 2 newly evolved of those 3 eukaryotic families then transferred back to prokaryotes via lateral gene transfer and, in addition, somehow each bacterial family then acquired their NfeD counterparts. As no eukaryotic NfeD homologs have been identified so far, they either have been lost without a trace in all eukaryotic families or have been acquired independently by most prokaryotic families – a rather unlikely event.

Our scenario explains the diversity of the eukaryotic SPFH members and the inability to find regions of synteny between the Flotillins, Prohibitins and Stomatins which would be probable if all eukaryotic families were derived from a single eukaryotic ancestor. In addition, it would be unnecessary to propose the convergent evolution of three eukaryotic and twelve prokaryotic families.

## Conclusion

In this study, we show that at least 4 (most likely 5) of the 12 prokaryotic SPFH protein subgroups that encompass almost 75% of all prokaryotic SPFH members are linked together by a similar operon structure and functional similarities (such as interaction with the FtsH protease). This suggests that they share a common origin. Their similarity to the three major eukaryotic SPFH families, as well as functional similarities suggests that the eukaryotic SPFH families originated from different prokaryotic SPFH families rather than from one. This explains the difficulties in obtaining a consistent phylogenetic tree for the eukaryotic SPFH members. In addition, lateral gene transfer is suggested as a source for the diverse occurrence pattern of different SPFH proteins and their accompanying Nfed partners.

## Authors' contributions

MH carried out the database searches and the sequence alignments, performed functional assays, and prepared proteins for NMR spectroscopy. AR participated in the design of the study and drafted the manuscript. HMM designed and supervised the NMR spectroscopic experiments and their analyses, and participated in the design of the study. CAW and AF performed the chemical shift assignment of YuaF and YqiJ. CAOS participated in the design of the study.

## Supplementary Material

Additional file 1 **Complete list of all bacterial species investigated with all identified SPFH family members and their accession numbers, list of all sequences used for the generation of the phylogenetic tree, their similarity to the generic SPFH HMM and their similarity to the respective subfamily HMMs.** Click here for file

Additional file 2 **Lowest and highest similarities of each subgroup to the SPFH hidden markov model (a) and to the subfamily specific HMMs (b).** Click here for file

Additional file 3 S**ize, domain structure and phylogenetic tree of SPFH subfamilies.** (a) Schematic representation of sizes and domain structure of all identified SPFH subfamilies, as well as the accompanying NfeD proteins. (b) Phylogenetic tree of prokaryotic SPFH proteins. The tree was created using the most diverse members of each subfamily, aligning them with ClustalW. Tree creation was performed with MEGA 4 (BLOSUM matrix and Neighbor joining algorithm, 1000 bootstrap replications). Click here for file

Additional file 4 **Phylogenetic tree of prokaryotic SPFH proteins.** The tree was created using the most diverse members of each subfamily, aligning them with ClustalW and tree creation was performed with MEGA 4 (BLOSUM matrix and Neighbor joining algorithm, 1000 bootstrap replications). Click here for file

Additional file 5 **Phylogenetic tree of representative SPFH 1 and 2 proteins (a) and phylogenetic tree of their accompanying NfeD proteins (b).** Click here for file
