# Evolution of prokaryotic SPFH proteins

**DOI:** 10.1186/1471-2148-9-22

**Published:** 2009-01-28

**Authors:** Markus Hinderhofer, Christina A Walker, Anke Friemel, Claudia AO Stuermer, Heiko M Möller, Alexander Reuter

**Affiliations:** 1 Department of Biology, Microbiology, University of Konstanz, Konstanz, Germany; 2 Department of Chemistry, NMR Spectroscopy, University of Konstanz, Konstanz, Germany; 3 Department of Biology, Neurobiology, University of Konstanz, Konstanz, Germany

After publication of this manuscript [1], we became aware that two citations that cover the prokaryotic SPFH1 proteins (here called paraslipins (SPFH1a) and eoslipins (SPFH1b)) [2] and the association of these SPFH1 proteins with NfeD proteins [3] which had been included in earlier versions of the manuscript had erroneously gotten lost during the corrections performed in the reviewing process.

We regret any inconvenience that these omissions might have caused and thank Prof. J.P.W. Young for bringing this matter to our attention.
