# Supplementary material for: Evolution of prokaryotic SPFH proteins
Source: BMC Evol Biol. 2009 Jan 12;9:10. doi: 10.1186/1471-2148-9-10 (PMC2636767; doi:10.1186/1471-2148-9-10)
Supplement: Additional file 2 — Lowest and highest similarities of each subgroup to the SPFH hidden markov model (a) and to the subfamily specific HMMs (b). [file 1471-2148-9-10-S2.doc]

**A: Highest and lowest E-value to the SPFH HMM**

|  | **highest E-Value** | **lowest E-Value** |
| --- | --- | --- |
| **SPFH1** | 3,50E-26 (YP_625112) | 8,60E-66 (YP_001306564) |
| **SPFH2** | 8,10E-01 (ZP_01692042) | 4,70E-38 (YP_146193) |
| **SPFH3a** | 1,50E-29 (AAO27216) | 5,10E-57 (ZP_01173251) |
| **SPFH3b** | 9,30E-32 (YP_353431) | 8,90E-57 (YP_666787) |
| **SPFH4** | 1,20E-07 (YP_755062) | 2,10E-55 (ZP_00531293) |
| **SPFH5** | 1,60E-01 (NP_469755) | 8,90E-05 (YP_144314) |
| **SPFH6** | 2,90E-13 (NP_813105) | 5,40E-37 (ZP_01645099) |
| **SPFH7** | 2,70E-41 (ZP_01105716) | 5,50E-52 (NP_269875) |
| **SPFH8** | 8,70E-01 (ZP_01361048) | 2,70E-07 (YP_371576) |
| **SPFH9** | 3,90E-09 (NP_441437) | 4,10E-47 (YP_172403) |
| **SPFH10** | 2,70E-03 (ZP_01692574) | 4,30E-22 (YP_001363859) |
| **SPFH11a** | 9,20E-21 (YP_001276552) | 4,40E-33 (ZP_00768346) |
| **SPFH11b** | 3,10E-14 (NP_867632) | 3,70E-26 (ZP_00768347) |
| **SPFH12** | 1,50E-06 (P_001243001) | 5,50E-30 (NP_485009) |

**B: Highest and lowest E-values to the subgroup-specific HMMs**

|  | **AC** | **Score** | **E value** |
| --- | --- | --- | --- |
| Spfh1a | NP_415022  YP_814873 | 670.50  300.90 | 3.70E-200  7.00E-89 |
| SPFH1b | YP_582959  YP_591605 | 6.56E+02  5.41E+02 | 2.90E-196  1.00E-161 |
| SPFH2a | NP_390979  NP_488568 | 912.7  487 | 1.10E-273  1.60E-145 |
| SPFH2b | NP_716997  ZP_01692043 | 1237.2  50 | 0.00E+00  1.20E-17 |
| SPFH2c | ZP_01060752  NP_867388 | 1535.2  1338.3 | 0.00E+00  0.00E+00 |
| SPFH3a | YP_286178  YP_075806 | 752  262 | 1.10E-224  3.40E-77 |
| SPFH3b | NP_418596  YP_709646 | 627.7  328.2 | 2.70E-187  3.90E-97 |
| SPFH4 | NP_906359  YP_755062 | 425.8  89.8 | 8.40E-127  1.20E-25 |
| SPFH5 | ZP_01724963  ZP_01853076 | 893  657.9 | 5.60E-268  3.20E-197 |
| SPFH6 | YP_146236  ZP_00372642 | 687.7  513.8 | 5.40E-206  1.20E-153 |
| SPFH7 | ZP_01105716  ZP_01772136 | 770.9  600.3 | 2.10E-231  4.80E-180 |
| SPFH8 | ZP_01058574  YP_243215 | 824.7  278.8 | 4.30E-247  9.00E-83 |
| SPFH9 | NP_772269  YP_001139885 | 1152.1  174.1 | 0.00E+00  1.20E-51 |
| SPFH10 | YP_001363859  ZP_01054906 | 1203.5  659.3 | 0.00E+00  3.60E-198 |
| SPFH11a | ZP_00768346  YP_828275 | 868.2  784.1 | 3.20E-261  6.40E-236 |
| SPFH11b | ZP_01529549  NP_867632 | 801.5  705.7 | 3.80E-241  2.50E-212 |
| SPFH12 | YP_383057  ZP_00567713 | 1547.6  905.2 | 0.00E+00  3.50E-272 |
