# Supplementary material for: Evolution of prokaryotic SPFH proteins
Source: BMC Evol Biol. 2009 Jan 12;9:10. doi: 10.1186/1471-2148-9-10 (PMC2636767; doi:10.1186/1471-2148-9-10)
Supplement: Additional file 5 — Phylogenetic tree of representative SPFH 1 and 2 proteins (a) and phylogenetic tree of their accompanying NfeD proteins (b). [file 1471-2148-9-10-S5.ppt]

## Slide 1
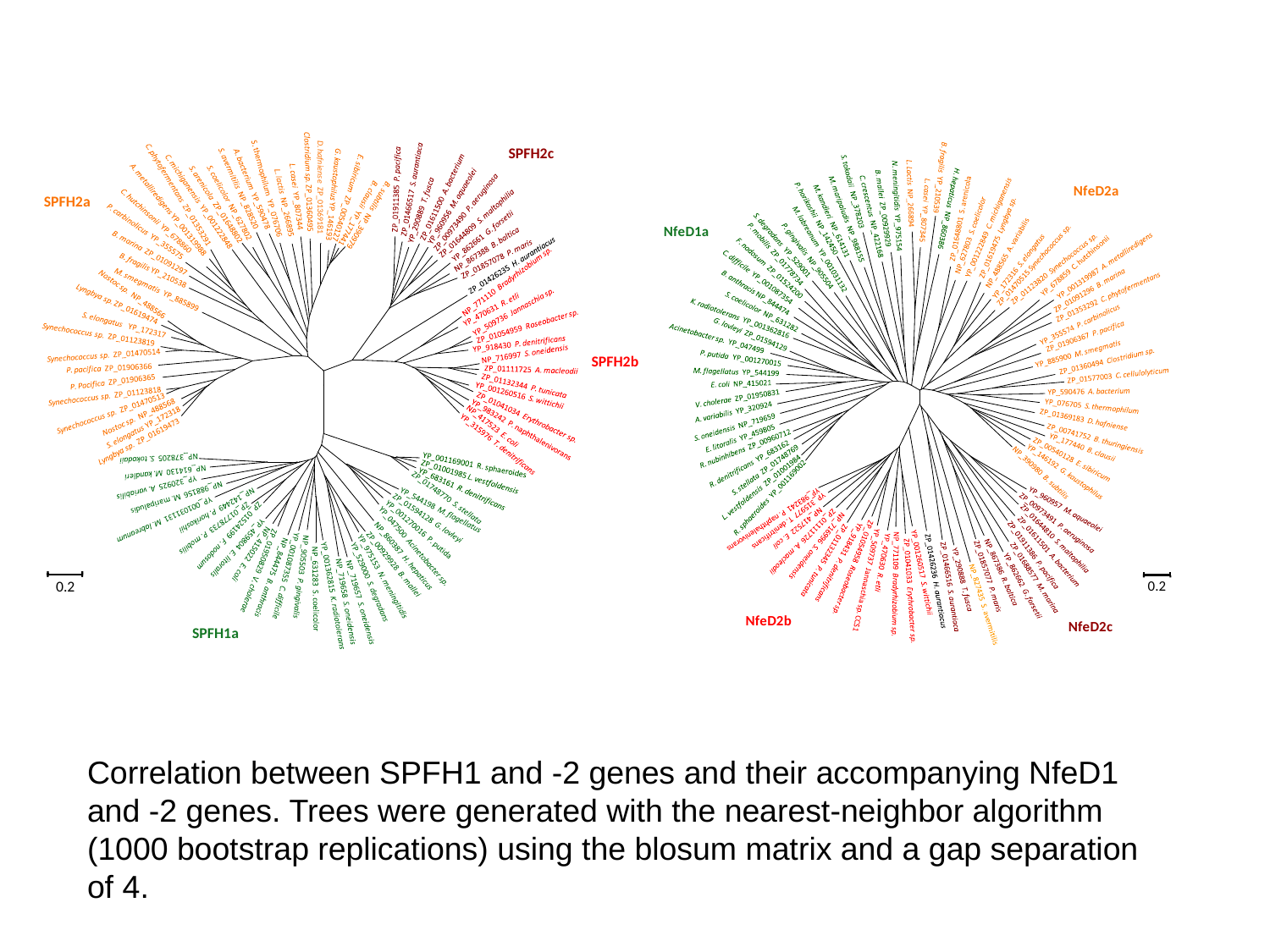

Correlation between SPFH1 and -2 genes and their accompanying NfeD1 and -2 genes. Trees were generated with the nearest-neighbor algorithm (1000 bootstrap replications) using the blosum matrix and a gap separation of 4.
